# Clinical Neuropathology mini-review 6-2015: PD-L1: emerging biomarker in glioblastoma? 

**DOI:** 10.5414/NP300922

**Published:** 2015-10-26

**Authors:** Matthias Preusser, Anna S. Berghoff, Wolfgang Wick, Michael Weller

**Affiliations:** 1 Department of Medicine I and Comprehensive Cancer Center CNS Unit, Medical University of Vienna, Vienna, Austria,; 2 Neurology Clinic, Heidelberg University Medical Center and Neurooncology Program, National Center for Tumor Diseases Heidelberg, Heidelberg, Germany, and; 3 Department of Neurology, University Hospital and University of Zurich, Zurich, Switzerland

**Keywords:** glioblastoma, PD-L1, PD-1, immune checkpoint, biomarker, prognosis, predictive

## Abstract

Programmed death 1 (PD-1, CD279) and programmed death ligand 1 (PD-L1, CD274) are involved in generating tumor-associated immunosuppression by suppression of T-cell proliferation and interleukin 2 (IL-2) production and immune checkpoint inhibitors targeting these molecules are showing compelling activity against a variety of human cancers. PD-L1 expression has shown a positive association with response to PD-1 inhibition in non-central nervous system (CNS) tumors, e.g., melanoma or non-small cell lung cancer, and is discussed as a potential predictive biomarker for patient selection in these tumor types. This review summarizes current knowledge and potential clinical implications of PD-L1 expression in glioblastoma. At present, the following conclusions are drawn: (a) functional data support a role for PD-1/PD-L1 in tumor-associated immunosuppression in glioblastoma; (b) the incidence of PD-L1-expressing glioblastomas seems to be relatively high in comparison to other tumor types, however, the reported rates of glioblastomas with PD-L1 protein expression vary and range from 61 to 88%; (c) there is considerable variability in the methodology of PD-L1 assessment in glioblastoma across studies with heterogeneity in utilized antibodies, tissue sampling strategies, immunohistochemical staining protocols, cut-off definitions, and evaluated staining patterns; (d) there are conflicting data on the prognostic role and so far no data on the predictive role of PD-L1 gene and protein expression in glioblastoma. In summary, the ongoing clinical studies evaluating the activity of PD-1/PD-L1 inhibitors in glioblastoma need to be complemented with well designed and stringently executed studies to understand the influence of PD-1/PD-L1 expression on therapy response or failure and to develop robust means of PD-L1 assessment for meaningful biomarker development.

## Introduction 

As early as 1863, Rudolf Virchow reported inflammatory infiltrates in tumor tissues and suggested an important link between cancer and the immune system [1]. Indeed, the interaction of tumor cells and immune cells has been confirmed as a major determinant of neoplastic disease and the ability of cancer cells to evade destruction by the immune system is today considered a hallmark of cancer [2]. Cancer-associated immunosuppression is mediated by various molecules and signaling pathways. Among these, immune checkpoint molecules including the programmed death 1 (PD-1) receptor and its ligands, programmed death ligand 1 (PD-L1) and programmed death ligand 2 (PD-L2), have emerged as important factors involved in immune evasion by tumor cells and monoclonal inhibitors of this signaling pathway show impressive therapeutic responses and favorable safety profiles across a variety of human cancers such as melanoma, lung cancer, renal cell cancer, bladder cancer, and others [3, 4]. A vast number of clinical trials in many tumor types are being launched and immune modulation by checkpoint inhibition is emerging as novel and important treatment paradigm in oncology. 

## The discovery of PD-1 and PD-L1 

**Figure 1.**
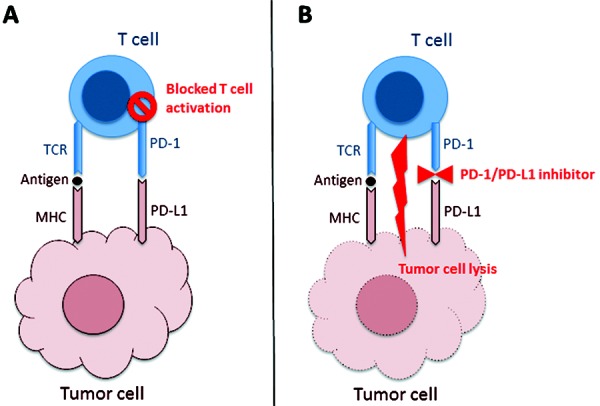
Cartoon showing the interaction of cytotoxic lymphocytes (T-cell) with tumor cells. A: Tumor cells present antigens on major histocompatibility complex (MHC) molecules to the T-cell receptor (TCR). T-cell activation is inhibited by an interaction of the co-inhibitory receptor programmed death 1 (PD-1; expressed on T-cells) with its ligand programmed death ligand 1 (PD-L1; expressed on tumor cells). B: Monoclonal antibodies targeting PD-1 such as nivolumab or pembrolizumab or PD-L1 such as atezolizumab block the inhibitory PD-1/PD-L1 interaction and thus facilitate T-cell-mediated tumor cell lysis.

PD-1 was discovered in 1992 in an attempt to identify genes that induce programmed cell death (apoptosis) [5]. Subsequently, the ligands PD-L1 and PD-L2 of this receptor and their role in the negative regulation of autoimmune response were identified [6, 7, 8]. In 2002, the relevance of PD-1 in cancer pathobiology was first observed, as it was shown that the blockade of PD-1 signaling restored an effective anti-tumor immune response with increased lymphocyte attack on myeloma cells [9]. Presence of PD-1-positive lymphocytes and expression of PD-L1 and their importance for immune escape of tumor cells have since been shown in many different cancer types including glioblastoma and other brain tumors (Figure 1) [10, 11, 12, 13, 14, 15, 16, 17]. The regulatory mechanisms of PD-L1 overexpression are poorly understood so far, but it has been related to activation of signaling cascades such as signal transducer and activator of transcription 3 (STAT3), loss of phosphatase and tensin homolog (*PTEN*) gene, gene rearrangements of the PD-L1 gene *CD274* or mutations of the 3’-untranslated region (UTR) of the *CD274* mRNA and other molecular alterations [18, 19, 20, 21]. 

## Clinical activity of PD-1 and PD-L1 inhibitors in non-CNS tumors 

The PD-1-inhibiting monoclonal antibodies nivolumab and pembrolizumab have shown favorable activity and good tolerability in clinical trials and have been approved for use in metastatic melanoma (nivolumab, pembrolizumab) and lung cancer (nivolumab) [4]. Approvals in more indications are pending and a multitude of clinical trials in many cancer indications are ongoing and under development with these, but also with other drugs targeting PD-1 and PD-L1. Of particular relevance is that responses including complete responses to immune checkpoint inhibitors are durable in some patients, whereas other patients seem not to benefit at all. The main toxicities are autoimmune events such as enteritis and endocrinopathies. 

## PD-L1 as a potential biomarker in non-CNS tumors 

PD-L1 protein as assessed by immunohistochemistry has been shown to positively correlate with response to PD-1 targeting therapy in several studies on melanoma, lung cancer, and other tumor entities, thus making this parameter a potential predictive biomarker [22, 23, 24, 25]. A pivotal trial demonstrated objective responses only in PD-L1-expressing tumors treated with the anti-PD-1 antibody (36% vs. 0% in PD-L1-positive and PD-L1-negative tumors, respectively) [24]. However, some studies failed to show a predictive value of PD-L1 expression and favorable responses have also been observed in considerable fractions of patients with PD-L1-negative tumors. Thus, controversial discussions around the feasibility of using PD-L1 as a marker for patient selection continue [26]. Ongoing research is being conducted to identify which patients with PD-L1-negative tumors respond to PD-1/PD-L1 treatment, and other immune-related factors such as tumor-infiltrating immune cells or other immune checkpoint molecules (e.g., PD-L2, another ligand of PD-1) are explored as candidate biomarkers. The issue is complicated by a lack of commonly accepted test methodologies for assessment of PD-L1 status, as a multitude of antibodies, staining protocols, readout methods, and cut-off definitions are being used in different studies. Furthermore, the sampling time point of the tissue samples used for PD-L1 expression analyses differed between studies, as in some studies archive tissue retrieved a considerable time before the initiation of the immune checkpoint therapy were utilized, while other investigations performed biopsies of target lesions at study entry [25]. However, the immune microenvironment of a given tumor might change over time, across localizations and importantly during systemic therapies as well as radiotherapy. In addition, studies varied with regard to the cell types evaluated for PD-L1 expression. Most studies concentrated on the membranous PD-L1 expression of viable tumor cells, while emerging data suggest a potential role of PD-L1 expression on circulating or tumor infiltrating immune cells such as macrophages or lymphocytes [27, 28]. Recently, overall mutational load, neoantigen load, and expression of cytolytic markers in the tumor microenvironment were significantly associated with response to immune checkpoint inhibitors in melanoma and lung cancer [29, 30, 31]. 

## PD-L1 in glioblastoma: current knowledge 

Glioblastoma, the most common primary brain tumor of adults, is characterized by poor survival rates and current therapy encompasses neurosurgical resection and adjuvant radiochemotherapy [32, 33]. Targeted agents have failed to show survival benefits so far and novel treatment approaches are urgently needed. Glioblastoma has long been recognized for its ability to generate an immunosuppressive milieu by upregulation of factors such as transforming growth factor (TGF)-β and indoleamine 2,3 dioxygenase (IDO) [19, 34, 35]. PD-1 and PD-L1 have also been implicated in immune escape of glioblastoma and we summarize here the current knowledge on these molecules in glioblastoma with a special focus on the potential role as biomarker for clinical patient outcome [15, 36, 37]. 

### Pre-clinical and functional data 

PD-L1 produced by glioma cell lines was reported to affect T-cell activation and decrease the production of lymphocytic interferon-γ and interleukins (IL) 2 and 10 [36, 38]. Expression of PD-L1 correlates with malignancy and inhibits CD4^+^ and CD8^+^ T-cells via PD-1 [36]. Furthermore, glioma cells increased PD-L1 expression in circulating monocytes and tumor-infiltrative macrophages by IL-10 signaling [37]. PD-L1 expression has also been reported on microglial cells in human glioblastoma specimens and microglia have been shown to block T-cells via PD-1/PD-L1 signaling in models of (auto-)inflammatory CNS diseases [10, 39]. PD-1 inhibition led to antitumor responses and increased survival in several studies on animal models of glioma [34, 40, 41]. Another member of this protein family, PD-L3 (B7-H3) may also exert relevant immunosuppression in glioma and is involved in infiltrative growth [42]. 

### PD-L1 protein expression in human tissue samples 

The available studies that have addressed the rate and extent of PD-L1 protein expression in glioblastoma are heterogeneous with differences in sample size, use of different tissue sampling strategies, use of different antibodies and staining protocols, use of different evaluation schemes for staining patterns, and use of different cut-offs (Table 1) [10, 12]. The considerable methodological differences between the studies limit their comparability. 

**Rate of PD-L1-expressing glioblastomas**

The reported rates of patients with glioblastomas with any PD-L1 protein expression on tumor cells were 61% (glioblastoma, not otherwise specified) in the study by Nduom et al. [10, 12] and 88% (newly diagnosed glioblastoma) and 72% (recurrent glioblastoma) in our study. One of several relevant methodological differences (Table 1) between these studies is the use of full histological slides in our and tissue microarrays in the study by Nduom et al. [12]. Tissue microarrays use smaller (in this case 1 mm) tumor tissue samples and are thus more prone to sampling bias than studies on full slides. We observed a patchy pattern of PD-L1 expression with positive and negative tumor areas on full slides. The lower percentage of PD-L1-positive glioblastomas reported by Nduom et al. [12] may be a result of a sampling error, i.e., false negative cases in which the tissue sample used for the tissue microarray represented PD-L1-negative areas of heterogeneous tumors with PD-L1 expression in other, not sampled tumor parts. Another plausible explanation for differing results between the studies is the use of different antibodies and immunostaining protocols. The results between the two studies are therefore numerically different, but not necessarily conflicting and jointly show that glioblastoma has a higher rate of PD-L1-positive cases than other tumor types [17]. 

**PD-L1 protein expression patterns**

**Figure 2.**
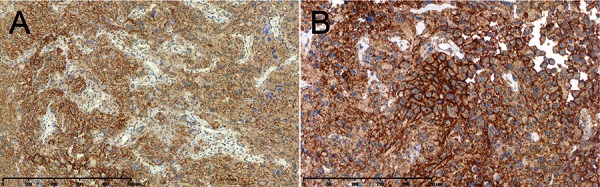
PD-L1 expression patterns in glioblastoma. A: Diffuse/fibrillary tumor parts show diffuse PD-L1 immunostaining of the tumor matrix. B: Interspersed epithelioid tumor cells show membrane-bound PD-L1 expression. A and B: Anti-PD-L1 immunostaining using antibody 5H1 as described by Berghoff et al. [10].

We found membrane-bound PD-L1 expression on interspersed epithelioid glioblastoma cells in 37.6% of newly diagnosed and 16.7% of recurrent glioblastoma cases (Figure 2) [10]. In addition, we observed patchy distribution of diffuse/fibrillary PD-L1 expression throughout the glioblastoma tissue of variable extent in 84.4% of newly diagnosed and 72.2% of recurrent glioblastomas. We consider the diffuse/fibrillary staining pattern to indicate PD-L1 expression on the delicate and intermingled tumor cell processes that form the pathognomic neurofibrillary matrix of diffuse astrocytic gliomas. This particular histomorphological feature of glioblastoma is distinct from histological appearances of other tumor types such as melanoma and carcinomas and therefore necessitates development of specific evaluation criteria for readout of immunohistochemical PD-L1 stainings. Nduom et al. [12] considered membrane-bound PD-L1 staining only and did not report on diffuse/fibrillary PD-L1 expression. Using a quantitative evaluation method they described a median percentage of glioblastomas with membrane-bound PD-L1 expression on tumor cells of 2.77% (range 0 – 86.6%). Interestingly, they additionally observed PD-L1-positive tumor-infiltrating lymphocytes in some cases, which may be a relevant finding, since PD-L1 expressing immune cells with potential predictive value have been described in other cancers, too [27, 28]. 

### Prognostic and predictive role of PD-L1 

**Prognostic role of PD-L1 gene expression**

The same two recent studies have evaluated the association of PD-L1 gene expression with patient survival in the TCGA dataset, with different results (Table 2) [10, 12]. Berghoff et al. [10] analyzed expression levels of the PD-L1 (*CD274*) gene of 446 patients using level 2 Agilent microarray gene expression data and found no correlation with patient outcome. In contrast, Nduom et al. [12] used level 3 Illumina RNASeq data from 194 patients and observed a significant correlation of high PD-L1 expression with unfavorable prognosis. The effect was independent of patient age on multivariate analysis, but none of the two studies tested for interactions with the known strong prognostic molecular parameter, *O6-methylguanine methyl-transferase gene* (*MGMT*) promoter methylation status. The differences between the two analyses in the number of investigated patient samples and in gene expression data likely affect the comparability of the results. Of note, poor correlation of gene expression levels measured by the Agilent microarray and IlluminaRNASeq in the TCGA dataset has been documented previously [43]. Overall, the prognostic role of PD-L1 gene expression remains unclear and further studies in independent, optimally prospectively collected patient cohorts seem warranted. Interestingly, Berghoff et al. observed a correlation of PD-L1 expression with molecular glioblastoma subtypes [44] with a higher rate of PD-L1 high expressors among mesenchymal glioblastomas and lower rates in glioma-CpG island methylator phenotype (G-CIMP) and proneural glioblastomas [10]. A higher immunogeneicity of the mesenchymal glioblastoma subtype has been described and it remains to be seen whether glioblastomas with this molecular signature respond differently to therapeutic immune modulation [45, 46]. 

**Prognostic role of PD-L1 protein levels**

There are conflicting reports on the prognostic role of PD-L1 protein levels. Berghoff et al. [10] investigated the prognostic implications of PD-L1 expression in a retrospective series of 117 adult newly diagnosed glioblastoma cases. The established prognostic factors patient age, Karnofsky performance score, extent of neurosurgical resection and *MGMT* promoter methylation status showed the expected separation of prognostic cohorts in this series, thus showing the principal validity of this patient cohort for exploratory survival analyses. There was no correlation with overall survival times for presence or absence of diffuse/fibrillary or membranous PD-L1 expression. Of note, PD-L1 expression on neurons in the infiltrated cortex did not show a correlation with patient outcome, which is in contrast with a prior small study of 17 cases [47, 48]. 

Nduom et al. [12] performed a retrospective study on a tissue-micro array containing 1 mm samples of 99 glioblastoma cases. It was not specified whether these were newly diagnosed cases and data on other prognostically relevant factors such as extent of resection, Karnofsky performance status or *MGMT* promoter methylation status were not reported. Using the median of 2.77% PD-L1 positive glioblastoma cells they did not find a significant correlation with patient outcome (p = 0.066). However, using a 5% cut-off in a secondary analysis, patients with high PD-L1 expression had significantly shorter survival times (p = 0.086). 

The considerable differences in applied methodology between the studies (Table 1) and their inherent limitations of retrospective and uncontrolled studies leave the prognostic role of PD-L1 protein expression unclear and further studies with adequate statistical power need to be conducted to resolve this open question. 

**Predictive role of PD-L1 expression**

So far, no clinical trials with PD-1 or PD-L1 inhibitors have been completed in glioblastoma and no data on the predictive role of PD-L1 expression for response to these drugs are available [15]. Testing of the predictive role of PD-L1 expression in glioblastoma samples collected in the ongoing trials will need careful consideration of assay validation, cut-off definitions and evaluation criteria to provide meaningful and clinically relevant results. 

## Summary and perspectives 

Emerging clinical studies document compelling antineoplastic activity of immune checkpoint inhibitors including those targeting PD-1/PD-L1 across several tumor types [4]. So far, no efficacy data for these drugs in human glioblastoma are available, but clinical studies are ongoing and will provide results in the near future. However, pre-clinical data from in-vitro and in-vivo studies and as well as data from human glioblastoma samples indicate that the PD-1/PD-L1 system is actively involved in creating tumor-associated immunosuppression in malignant glioma and make it a rational treatment target [13, 15]. The blood-brain/blood-tumor barrier is expected not to limit the influx of activated immune cells into the CNS and primary brain tumors and will hopefully not significantly compromise the activity of immune checkpoint inhibitors in gliomas, although proof of this hypothesis is lacking at the moment [15, 49]. Importantly, the rate of PD-L1-expressing glioblastomas seems to be relatively high in comparison to other tumor types, although the reported incidences of PD-L1-positive, albeit with widely varying cell numbers, glioblastomas vary and range from 61 to 88% [10]. This heterogeneity is likely related to differences in methodology of PD-L1 assessment across studies and investigations aiming at identification and validation of optimal test assays should be a priority to ensure meaningful biomarker development. Importantly, the particular histomorphology of glioblastoma with the pathognomonic gliofibrillary matrix, which is distinct from histological features of other tumor types such as melanoma and carcinomas, necessitates development of specific evaluation criteria for readout of immunohistochemical PD-L1 stainings. The prognostic role of PD-L1 gene or protein expression is unclear at the moment, as few studies have been conducted on this issue and have shown inconsistent results [10]. Furthermore, the predictive role of PD-L1 expression for response to monoclonal antibodies targeting PD-1/PD-L1 in glioblastoma remains to be elaborated in translational studies accompanying the ongoing clinical trials. Possibly, relevant cues for predictive markers will also be derived from other molecular characterizations such as overall mutational load, neoantigen load, or expression of cytolytic markers in the tumor microenvironment [29, 30, 31]. This association of a potentially immune activating mutational tumor and microenvironment may finally need to be included in biomarker assessments. 

## Acknowledgment 

Antibody 5H1 was kindly provided by Dr. Lieping Chen, Yale University. 

## Conflicts of interest 

MP has received honoraria and research support from Bristol-Myers Squibb, Merck Sharp & Dohme, Boehringer Ingelheim, GlaxoSmithKline, Mundipharma, and Roche. ASB has received honoraria and travel support from Bristol-Myer Squibb, Amgen, and Roche. WW has received research grants from Apogenix, Boehringer Ingelheim, Eli Lilly, immatics, MSD, and Roche as well as honoraria for lectures or advisory board participation from MSD and Roche. MW has received research grants from Acceleron, Actelion, Alpinia Institute, Bayer, Isarna, MSD, Merck & Co, Novocure, PIQUR, and Roche and honoraria for lectures or advisory board participation or consulting from Celldex, Immunocellular Therapeutics, Isarna, Magforce, MSD, Merck & Co, Northwest Biotherapeutics, Novocure, Pfizer, Roche, and Teva. 

**Table 1.** Comparison of immunohistochemical methods and results of PD-L1 protein expression analysis and its prognostic role in two recent studies [10, 12].

Parameter/study		Berghoff et al. [10]	Nduom et al. [12]
Study design		Retrospective	Retrospective
Sample size		117	99
Assay		Immunohistochemistry	Immunohistochemistry
Tissue samples		Full slides	Tissue microarray
Antibody		5H1	EPR1161(2)
Staining patterns		Membranous on tumor cells	Membranous on tumor cells
		Diffuse/fibrillary in tumor matrix	On tumor-infiltrating lymphocytes
Cut-offs	None	15.4% of cases	Not reported
	> 1%	Not reported	60.6% of cases
	> 5%	Not reported	38.3% of cases
	> 25%	Not reported	17% of cases
	> 50%	Not reported	50% of cases
	Diffuse/fibrillary 1 – 25%	15.4% of cases	Not reported
	Diffuse/fibrillary 26 – 50%	25.6% of cases	Not reported
	Diffuse/fibrillary 51 – 75%	33.3% of cases	Not reported
	Diffuse/fibrillary 76 – 100%	10.3% of cases	Not reported
	Membranous < 5%	62.4% of cases	Not reported
	Membranous 5 – 100%	37.6% of cases	Not reported
Significant correlation of overall survival with known prognostic parameters	Extent of resection	Yes	Not reported
	Patient age	Yes	Not reported
	Karnofsky performance score	Yes	Not reported
	*MGMT* promoter methylation status	Yes	Not reported
Significant correlation of PD-L1 protein expression with overall survival	Diffuse/fibrillary PD-L1 expression present vs. not present	No (p = 0.921)	Not reported
	Membranous PD-L1 expression ≤ 2.77% vs. > 2.77%	Not reported	No (p = 0.066)
	Membranous PD-L1 expression < 5% vs. ≥ 5%	No (p = 0.724)	Yes (p = 0.0086)

**Table 2.** Comparison of the TCGA analyses of PD-L1 gene expression and is prognostic role in two recent studies [10, 12].

Parameter/study		Berghoff et al. [10]	Nduom et al. [12]
Sample size		446	194
Assay		Agilent microarray	Illumina RNASeq
Significant OS correlation of PD-L1 gene expression	Univariate	No (HR = 1.038, 95% CI 0.9553 – 1.368, p = 0.144)	Yes (HR = 1.54, 95% CI 1.05 – 2.28, p = 0.0231)
	Multivariate (PD-L1, age)	No (HR = 1.036, 95% CI 0.8702 – 1.232, p = 0.694)	Yes (HR = 1.52, 95% CI 1.03 – 2.25, p = 0.0343)
